# Health and economic effects on patients with type 2 diabetes mellitus in the long run: predictions for the Chilean population

**DOI:** 10.1186/s13098-022-00928-4

**Published:** 2022-10-26

**Authors:** Manuel Antonio Espinoza, Tomas Abbott, Alvaro Passi, Carlos Balmaceda

**Affiliations:** Departamento de Salud Pública, Unidad de Evaluación de Tecnologías Sanitarias, Pontificia Universidad Católica de Chile, Diagonal Paraguay, Piso 2, 362 Santiago, Chile

**Keywords:** Diabetes type 2, Long-term, Costs, Health outcomes, Population predictions, Cardiovascular risk factors

## Abstract

**Background:**

Diabetes is associated to a high financial and disease burden, explaining a large proportion of expenditure of the health system in one year. The purpose of this study was to estimate long-term costs and health outcomes of recently diagnosed patients with type 2 diabetes in Chile.

**Methods:**

Cost and consequence study based on mathematical discrete event simulation (DES) model. We modelled expected costs (USD) and quality-adjusted life-years (QALYs) from diagnosis to death (or the age of 95) of a hypothetical cohort of 100,000 incident cases, simulated based on the Chilean National Health Survey 2018. The incidence of twelve complications was estimated assuming the hazard functions provided by the United Kingdom Prospective Diabetes Study. We explore heterogeneity across patients based on their baseline risk covariates and their impact on costs and QALYs.

**Results:**

The expected cost and QALY of a recently diagnosed type 2 diabetes patient in Chile were USD 8660 and 12.44 QALYs. Both costs and QALYs were independently determined by baseline risk and the patient's life expectancy from the diagnosis. Length of life since diagnosis showed the major impact on costs (5.2% increase for every additional year). Myocardial infarction was the most frequent complication (47.4%) and the most frequent cause of death.

**Conclusion:**

Diabetes type 2 determines a significant expenditure of the health system and substantial health losses. Although the control of cardiovascular risk factors and the metabolic control of the disease, both have an important impact on costs and outcomes, the main impact is achieved by postponing the age of onset of the disease.

**Supplementary Information:**

The online version contains supplementary material available at 10.1186/s13098-022-00928-4.

## Introduction

Type 2 Diabetes (DM2) is one of the health problems that has grown fastest in the world, with an estimated increase from 2.8% in the year 2000 to 4.4% in 2030, leading to a population of affected adults that would increase from 171 to 366 million [1]. DM2 has also been associated with a significant high financial burden on healthcare systems, mainly due to its complications in the long run [2]. They include end-stage renal disease, cardiovascular disease, neurological and ophthalmological disorders. Hence, DM2 not only determines many resources spent by the healthcare system but it is also responsible for very significant impairment in health-related quality of life (HRQoL) because of the above-mentioned complications.

In Chile, the prevalence of DM2 increased from 4.2 to 12.6% in the adult population between 2003 and 2017, respectively [3]. Furthermore, the burden of DM2 has been estimated at 72,230 years of life lost due to disability [4], which corresponds to approximately 2% of the total burden of disease in the country. However, despite this high prevalence, the proportion of adults with DM2 who are properly and effectively managed only reaches 34% [5]. In addition, in Chile overweight and obesity reached the highest prevalence documented for this country in 2017, 39% and 22%, respectively. These results suggest that DM2 will continue to increase in the coming years with the progressive ageing of the population (life expectancy: 81.2 years for women and 77.3 years for men) [6], which will impact either on population health outcomes or the financial burden on the healthcare system.

However, despite the vast evidence supporting the rationale of preventing long-run complications in DM2, healthcare systems often deal with budgetary myopia of the political system, i.e. authorities are more willing to allocate resources to activities that produce benefits in the short-run rather than in the long-term [7]. Consequently, resources are allocated to more severe problems, postponing preventive actions, which may ultimately affect the sustainability of the system. Indeed, most research has been focused on producing estimates of the cost of diabetes in the short-run [8–10]. For example, a recent review reported the lack of studies modelling long-term economic and health effects of diabetes in low- and middle-income countries [11]. The purpose of this study was to estimate the expected cost and health outcomes in the long run for recently diagnosed diabetic patients in Chile. We believe that the characterization of financial consequences, as well as health outcomes throughout patients’ lives, incorporate an essential element to the health policy analysis and it may serve as support for decision-making in DM2-related policies.

## Methods

A discrete event simulation (DES) model was developed de novo to estimate the expected time through which an individual newly diagnosed with DM2 develops different complications of the disease throughout his/her life. The DES model was informed by two data sources to estimate the time to event of complications: the hazard of every complication as a function of individual baseline risk characteristics; and the baseline risk characteristics of the Chilean population recently diagnosed with DM2. Hazard functions used for this model correspond to the Outcomes Model 2 of the United Kingdom Prospective Diabetes Study (UKPDS) [12]. The referred report provides functions for 12 complications estimated from this British cohort followed for 30 years. This was the longest and most robust publicly available data we had access to inform our model. On the other hand, the baseline risk characteristics of adults with recently diagnosed DM2 were determined using the data reported by the nationally representative Chilean Health Survey (CHS) [13]. From this, 41.35% of the adults who had baseline fasting blood glucose levels ≥ 126 mg/dL, did not know or reported not having diabetes. We assumed that this subset of patients represents adequately new cases of DM2.

The CHS for this subgroup of patients contained a significant amount of missing data for informative covariates, which might lead to a loss of 40% of the sample. We used multiple imputation techniques to sort out this problem. We run 20 iterations using the fully conditional specification in a multivariate imputation by a chained equation for haemoglobin A1c (HbA1c), low-density-lipoprotein cholesterol (LDL), high-density-lipoprotein cholesterol (HDL), and Haemoglobin variables.

To run the DES model, we used bootstrap as a sampling method to simulate 100,000 patients from the above-mentioned subsample. Conceptually, every patient entered the model and transited until one or more complications occurred. The time and the occurrence of every event depend on the hazard equations provided by the UKPDS study [12]. Alongside the exercise, we recorded either cost incurred because of each complication or losses in health-related quality of life over time, for every simulated patient.

In this study, we modelled the expected time of suffering every complication independently based on the baseline risk profile. Then, estimates of time to event for every complication were ordered from the earliest to the latest for every patient, which allowed us to estimate their course of complications across time. Thus, after one event appears, the mortality equations were recalculated, representing the change in the risk profile of each patient conditional to the event occurrence. If death happens before any complication, we stopped recording costs and years. The time horizon for this study for the *i*th subject was a lifetime or the age of 95 years old. We defined this cut-off because according to the national statistics, the remaining alive population was less than 0.15%.

Additionally, we modelled the number of moderate and severe hypoglycaemia events using the model built by Balmaceda et al. [14], and the risk profile data of recently diagnosed Chilean diabetic patients. Using 100,000 simulations from the hypoglycaemia model, a two-hurdle model was run to obtain equations for moderate and severe events, which it was incorporated into the DES model.

The costs related to the treatment and complications of the disease were incorporated to estimate the expected total cost in the long run. They included the costs of the baseline treatment of DM2 and those incurred due to the complications captured in the model. Costs were measured in Chilean pesos (CLP) and converted into USD dollars using an exchange rate of USD 1 = CLP 680. The perspective represents the Chilean public healthcare system. For every complication, we estimated an expected cost using the basket of services guaranteed by the Chilean universal health benefit plan [15, 16]. The baskets include healthcare services for diagnostic, treatment, follow-up, and rehabilitation with a defined set of consultants, laboratory tests, images, surgeries, and drug therapy, among others [15, 16]. All costs were adjusted by the variation of the consumer price index, with the value of January 2020. Because the DES model transits in time continuously [17], costs (which are on an annual basis) will consider the fraction of time in which the treatment (or complication) was received (or suffered) by the patient over his/her life. For example, if one person develops end-stage kidney disease in the year 15 and 2 months, costs accumulate from that exact moment onwards. A discount rate of 3% per year was only used for costs according to the Chilean Ministry of Health guideline, to express expected cost in present value [18].

We incorporated HRQoL into the economic model using the quality-of-life scores (often called utility values) to estimate quality-adjusted life-years (QALYs). Disutility values were considered for every complication suffered by patients over their lives. These values were obtained from the literature and are presented in Additional file 2.

Finally, heterogeneity was explored through subgroup analysis to characterize the economic and health effects of different risk profiles. These subgroups were defined using HbA1c, Body Mass Index (BMI), and Systolic Blood Pressure (SBP) at two cut-offs each. Determinants of the expected cost and QALYs were explored using linear models (Generalized Linear Models (GLM) and Ordinary Least Squares). The coefficient for the cost regression must be interpreted as semi-elasticities because they were obtained through a GLM with gamma family function and log link function. We explored the independent effect of age at diagnosis, time from diagnosis to death, BMI, SBP and HBA1c as continuous variables. The model was developed with TreeAge Pro 2018® software and data analysis was made with STATA 14®.

## Results

The DES model produced 100,000 simulated recently diagnosed patients, with similar baseline characteristics to those reported in the original CHS. The comparison between these baseline covariates is presented in Additional file 2. Table 1 summarises the restricted mean survival time, the median time, the expected time to the event in the subset of patients who suffered the event, and the cumulative incidence. Our findings show that 98.74% of the cohort died before or at the age of 95 years old, 64.19% died due to non-diabetes-related causes and the remaining 34.55% died because of one event attributable to DM2. Myocardial infarction was the most frequent cause of death related to DM2. 19.19% of the cases died because of the first myocardial infarction (MI) and 6.90% died due to a second MI. The next more recurrent causes of death were the first episode of stroke (3.92%) and Ischemic Heart Disease (IHD) (2.28%). As expected, blindness was the only event that was not associated with mortality.

**Table 1:** Restricted mean survival time, median, conditional expected time and cumulative incidence for complications attributable to diabetes and death

Outcomes (time to event)	RMST (years)	Median (SE)	Conditional mean time (years)	Cumulative incidence of the event
Time to death	15.6	13.6 (0.034)	–	0.9874
Time to death due to other causes	20.6	19.9 (0.034)	–	0.6419
Time to death/MI	10.6	8.45 (0.038)	–	0.1919
Time to death/second MI	17.6	16.4 (0.097)	–	0.0690
Time to death/stroke	13.5	12.1 (0.081)	–	0.0392
Time to death/IHD	15.1	13.4 (0.115)	–	0.0228
Time to death/DND	11.7	9.21 (0.145)	–	0.0123
Time to death/second stroke	20.3	19.6 (0.282)	–	0.0054
Time to death/amputation	22.1	21.4 (0.274)	–	0.0026
Time to death/second amputation	22.1	20.2 (0.507)	–	0.0019
MI	24.6	16.5 (0.032)	6.7	0.4745
Heart Failure	42.0	N/A	9.0	0.1534
Stroke	46.1	N/A	8.7	0.1104
Second MI	44.8	N/A	16.6	0.0894
IHD	48.1	N/A	9.1	0.0749
DND	51.6	N/A	5.1	0.0445
Blindness	51.7	N/A	6.3	0.0429
Amputation	53.3	N/A	17.1	0.0119
Second Stroke	53.7	N/A	19.3	0.0092
Second Amputation	54.2	N/A	20.2	0.0040

SE, Standard Error; MI, Myocardial Infarction; DND, Diabetic Nephropathy in Dialysis; IHD, ischemic heart disease; N/A: Not available; RMST: Restricted Mean Survival Time. The conditional median time corresponds to the median time given the patient has survived until the occurrence of the event. Results were obtained from the survival analysis over the microsimulated population for all the outcomes specified as diabetes complications. The median is the median time until the 50% of the population have developed the event

The restricted mean survival time (RMST), which represents the expected time measured from the diagnosis to death or up to the age of 95 years old, was estimated at 15.6 years (Table 1). Taking the average age of the population of 58 years old, this is equivalent to a life expectancy at the diagnosis of 73.6 years old. The subset of patients who died because of other causes showed an RMST of 20.6 years, which suggests that death due to complications of DM2 occurs -on average- at least 5 years earlier than people who die because of other causes. Accordingly, the most frequent diabetes-related causes of death showed relatively low RMST. For example, RMST due to MI was 10.6 years and RMST due to stroke was 13.5 years. Estimates of the median time, which measures the time to which the probability of survival beyond that time is 50%, were consistent with the results of RMST. The largest median time to death was observed for the subset of non-diabetes-related causes.

Table 1 also shows the estimates for complications of DM2. The most frequent complication observed in the lifetime of patients was MI. Almost half of the patients with DM2 (47.45%) will present this complication before dying. This complication is followed by heart failure (15.34%), stroke (11.04%) and a second MI (8.9%). We also found that less than 5% of patients will present diabetic nephropathy in dialysis (DND) (4.45%), blindness (4.29%) and amputation (1.19%).

For the expected time to event or complication, we also present the conditional mean, which restricts the analysis to those individuals who were alive when the event occurred. Thus, while the RMST for MI was 24.6 years, which is the mean time regardless the patient was alive or dead, the conditional mean was only 6.7 years. We claim that the conditional mean provides an easier and more useful interpretation. For example, the expected time to suffer MI in those patients who survived until the event occurred was 6.7 years. The complication with the lowest conditional mean was DND, indicating that in the subgroup of patients who will suffer DND, this complication is expected to happen shortly, specifically 5.1 years.

Table 2 shows the expected costs and QALYs for the entire patient population and subgroups constructed by a subset of variables representing different risk profiles. Our findings show that the cost the healthcare system expects to spend on one recently diagnosed patient with DM2 over his/her life is USD 8660. In general, the cost is associated with the time patients stay in the model, i.e. the longer they live the higher the cost. It can also be observed that subgroups with higher risk die early, which implies that they also present lower expected costs. On the other hand, the expected QALYs for the entire group of patients is 12.44 QALYs. Subgroups also show some variations with higher and lower estimates. For example, patients with HbA1c > 8 show 13.67 QALYs and patients with BMI > 30, 13.12 QALYs. Likewise, as in the case of costs, this variation seems to be associated with the time patients stay in the model.

**Table 2:** Expected and adjusted expected cost and QALYs. subgroup analysis

Subgroup	Expected cost (USD)	Expected QALY	Annual expected cost (USD)	Annual expected QALY	Mean age	Mean time in model	Proportion of the sample (%)
All patients	$8660	12.4412	$735	0.8065	58.0	15.51	100
HbA1_c_ > 8	$10,032	13.6773	$777	0.8042	54.49	17.08	48.70
HbA1_c_ > 9	$9948	13.3486	$784	0.8044	54.85	16.67	34.78
SBP > 130	$7984	10.9902	$794	0.8058	60.45	13.72	60.78
SBP > 160	$7177	8.4897	$917	0.8031	64.23	10.67	16.77
BMI > 25	$8866	12.7457	$732	0.8060	57.34	15.89	90.75
BMI > 30	$9020	13.1245	$735	0.8055	56.85	16.38	65.49
HbA1_c_ > 8 and SBP > 130	$9103	11.6253	$854	0.8031	57.91	14.55	29.08
HbA1_c_ > 9 and SBP > 130	$9494	12.1436	$851	0.8031	56.80	15.20	19.86
HbA1_c_ > 8 and SBP > 160	$8045	8.9691	$974	0.7992	61.95	11.32	9.28
HbA1_c_ > 9 and SBP > 160	$8799	9.5240	$1,001	0.8001	59.17	12.00	4.67
HbA1_c_ > 8 and BMI > 25	$10,164	13.9322	$778	0.8039	54.07	17.40	44.09
HbA1_c_ > 9 and BMI > 25	$10,060	13.5471	$788	0.8040	54.50	16.92	31.11
HbA1_c_ > 8 and BMI > 30	$9961	13.7358	$793	0.8035	54.79	17.17	32.96
HbA1_c_ > 9 and BMI > 30	$9580	12.7910	$816	0.8030	56.11	16.01	22.69
BMI > 25 and SBP > 130	$8065	11.1920	$785	0.8054	60.12	13.98	56.18
BMI > 30 and SBP > 130	$8342	11.5677	$796	0.8051	59.30	14.45	44.95
BMI > 25 and SBP > 160	$7241	8.5829	$904	0.8022	63.97	10.80	14.94
BMI > 30 and SBP > 160	$7344	8.6910	$927	0.8025	63.18	10.91	11.17
HbA1_c_ > 8 and SBP > 130 and BMI > 25	$9021	11.5946	$856	0.8028	58.03	14.52	26.34
HbA1_c_ > 9 and SBP > 130 and BMI > 25	$9349	12.0431	$854	0.8029	57.03	15.08	18.04
HbA1_c_ > 8 and SBP > 160 and BMI > 25	$7589	8.3803	$993	0.7988	63.13	10.60	8.39
HbA1_c_ > 9 and SBP > 160 and BMI > 25	$7968	8.3483	$1,049	0.7995	61.11	10.54	3.78
HbA1_c_ > 8 and SBP > 130 and BMI > 30	$9032	11.5038	$879	0.8029	58.23	14.41	21.75
HbA1_c_ > 9 and SBP > 130 and BMI > 30	$9387	11.9048	$874	0.8025	57.08	14.91	16.17
HbA1_c_ > 8 and SBP > 160 and BMI > 30	$7489	7.8011	$1063	0.8001	63.59	9.85	6.55
HbA1_c_ > 9 and SBP > 160 and BMI > 30	$7968	8.3483	$1049	0.7995	61.11	10.54	3.78

BMI, Body Mass Index; HbA1_c_, Glycated Haemoglobin A1_c_; SBP, Systolic Blood Pressure. Expected cost and QALYs obtained from the analysis of different risk subgroups within the micro-simulated cohort

Table 2 also shows annual expected costs and QALYs. The former corresponds to the average individual annual cost, which was estimated as the individual total cost divided by the time in the model. The latter was estimated using the same rationale and can be interpreted as the utility value that one patient is expected to experience for one year, on average during his/her life. Most subgroups showed an annual cost larger than the one estimated for the entire population. Furthermore, all subgroups presented lower annual QALYs estimates compared to the general patient population. These findings are consistent with the idea that higher risk explains more cost and fewer QALYs.

**Fig. 1:**
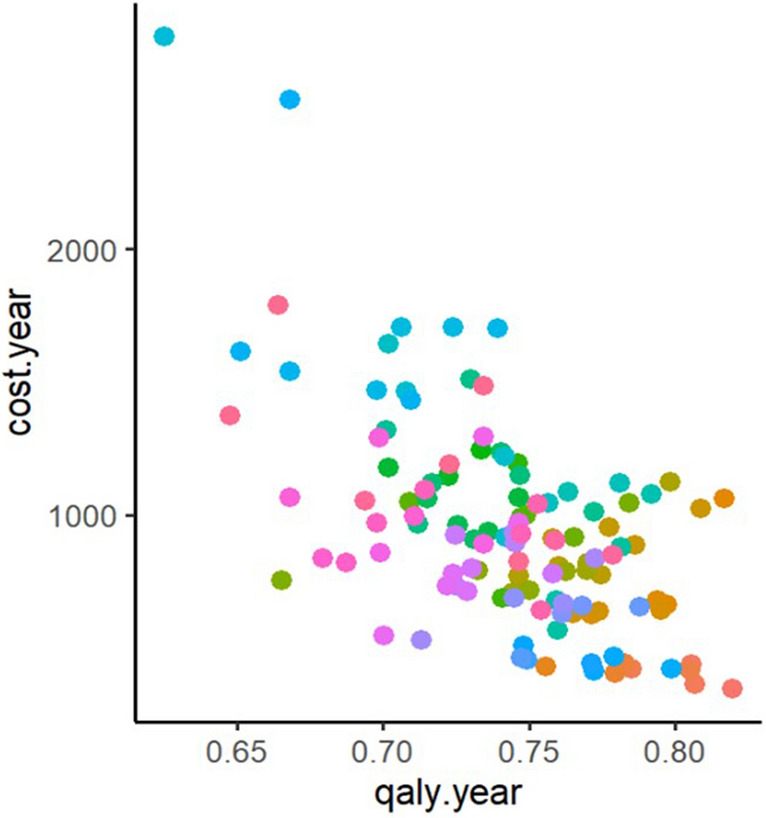
Association between annual cost and annual QALY among different subgroup specifications. *Colour points: corresponds to each of the 118 subgroups constructed based on all the possible combinations of consequences. Detailed subgroups estimates are available in Additional file 1

Besides examining baseline risk heterogeneity, we explored outcome-based heterogeneity. We estimated the costs and outcomes of subgroups defined by the number of complications every patient suffered throughout his/her life. Our results showed 118 different subgroups, which resulted from the combination of two or more complications attributable to diabetes. Figure 1 illustrates the relationship between the average annual cost and average annual QALY for every subgroup. We observed a clear negative relationship between these two estimates. Because higher QALYs are expected in subgroups with less baseline risk, these findings are coherent with the hypothesis that higher baseline risk is associated with higher cost.

To further explore these associations, we run multivariable regression models examining the effect of a set of baseline risk covariates (age, HbA1c, BMI, LDL and SBP) and time from diagnosis to death. These results are presented in Table 3. As expected, time from diagnosis to death was significantly associated with cost and QALYs. Hence, an increase of one unit in time is associated with an increase of 5.2% in cost. Likewise, all baseline risk covariates were directly and significantly associated with cost. Age also showed a significant but inverse association, i.e., each additional year at the time of diagnosis was associated with a 1.1% lower cost. These findings are consistent with the argument that the cost is partially explained by the time one person stays alive with diabetes, but also -and independently-because of the baseline risk at the time of diagnosis. The analysis of QALYs also showed that the time to death was significantly associated with more QALYs, which is consistent with the fact that longer life expectancy is associated with more QALYs. Likewise, age showed an inverse significant association with QALYs, which also relates to the idea that shorter life expectancy is associated with shorter QALYs. Interestingly, these two covariates were statically significant, which means that QALYs are determined in part by the age at which patients are diagnosed and -additionally and independently- by the time they stay alive. In contrast, baseline risk covariates showed a significant negative association with QALYs, meaning that the higher the baseline risk the lower the QALYs. This is consistent with the argument that higher baseline risk produces complications that affect HRQoL.

**Table 3:** Multivariable analysis of determinants of costs and QALYs

Covariate	Dependent variable: Cost (USD) Model GLM family(gamma) link (log)		Dependent variable: QALYs OLS	
	β Coefficient (SE)*	P-value	β Coefficient (SE)	p-value
Time from diagnosis to death (years)	0.0521803 (0.0003126)	< 0.0001	0.7937634 (0.0002864)	< 0.0001
Age (years)	− 0.011889 (0 0.0003061)	< 0.0001	− 0.0043533 (0.0002901)	< 0.0001
Glycosylated hemoglobin A1c (%)	0.0383068 (0.0010653)	< 0.0001	− 0.0117412 (0.001081)	< 0.0001
Systolic Blood Pressure (mmHg)	0.0019214 (0.0001038)	< 0.0001	− 0.0018695 (0.0001039)	< 0.0001
Body Mass Index (kg/m^2^)	0.0015645 (0.0004381)	< 0.0001	− 0.0020601 (0.0004291)	< 0.0001
LDL Cholesterol (mg/dL)	0.0028845 (0.000072)	< 0.0001	− 0.0006334 (0.0000675)	< 0.0001

*Coefficient of the GLM model must be interpreted as a semi-elasticity, i.e. for each unit increase in the independent variable, the dependent variable increases (if positive coefficient) β (*100) percent. GML: Generalized linear Models; OLS: Ordinary Least Squares; SE: Standard Error

## Discussion

Diabetes is a major public health problem that causes a high economic and disease burden, mainly due to its related complications. In Chile, like other jurisdictions across the globe, statistics in the last few years indicate that DM2, and its consequences, are increasing over time [19–21]. This study allowed us to estimate the effects of DM2 in the long run in the population of recently diagnosed patients with this condition and characterise the variability across different individuals based on their baseline risk covariates. Our results showed that the expected costs and QALYs of an adult with DM2 were USD 8660 and 12.44 QALYs. We also found that costs are explained by two independent elements: (i) the baseline risk which is associated with complications that demand healthcare resources; (ii) and the length of life from the diagnosis. The former relates to the higher expenditure on healthcare due to complications and the latter to the fact that because more time alive also consumes more resources. In terms of QALYs, our findings showed that either baseline risk or length of life from diagnosis showed a significant inverse association with QALYs. While the length of life contributes directly to the life years, baseline risk contributes to both, a decrease in HRQoL and length of life.

To our knowledge, this is the first modelling study in a low-and-middle-income country that reports the long-term outcomes and costs. One of the strengths of our study is that not only provides a characterization of heterogeneity in costs and outcomes and their magnitudes, but also a quantification of the potential impact of controlling parameters such as HBA1c, SBP, BMI and LDL on the cost and QALYs. For example, a reduction of 1% of the HbA1c is associated with a 3.8% reduction in costs. Likewise, this impact is also observed in HRQoL, which should be interpreted as the amount of disutility averted because reducing one unit of the baseline risk covariate. Although these magnitudes are informative, we are aware that cannot be claimed a hierarchy among those covariates. The fact that HBA1c showed the highest coefficient in the model, does not mean it has the highest impact, because it depends on the equivalence of the units of each covariate.

Although short-term cost estimates are useful because they inform the amount of money one health system must allocate to it in one year, we argue that long-term estimates complement the information for health policy decisions. These estimates can offer projections that may illustrate more clearly the relevance of the disease over time. For example, assuming an incidence rate of 6.9 per 1000 patient-years [23], we can calculate that an annual cohort of new DM2 patients in a population older than 15 years (96,880 patients) [24] determines a long-term expenditure of USD 839 million for the Chilean health system. This calculus assumes that patients will have access to the same services offered today, or -if new services are covered- they do not carry additional healthcare costs. Putting this estimate in context, this means that new DM2 patients in one year represent a long-term expenditure equivalent to 6.6% of the total annual public health expenditure of the country.

Our model also predicted that, among all complications attributable to DM2, MI was the most frequent event and the most frequent cause of death. This finding is consistent with previous literature that indicates that cardiovascular disease, and particularly, ischemic cardiac diseases are the most frequent complications [19, 25–28]. Furthermore, our results are also coherent with results of the UKPDS model [12] and mathematical models that have used the UKPDS as an input (25). Up to our knowledge, there is no study reporting an incidence estimate throughout the life’s patient. It is worth noting that our estimates are function of the baseline characteristics of the Chilean population, which may explain some differences with other studies where the baseline risk pool of the population differs.

One limitation of our model is that over 1% of the patients remain alive at the age of 95 years old, in circumstances that national statistics indicate that this proportion is only 0.15%. This lack of accuracy of our model at this stage may be explained by the general estimates of mortality provided in the lifetables which assume the same probability of death for all people above 85 years old. In addition, as described above, the risk equations we used to inform our model were obtained from a British cohort, which might have contributed to this lack of precision. Notwithstanding, we argue that this small difference in absolute terms does not determine significant changes in our results.

Another limitation of these results relates to the generalizability to other jurisdictions. We are aware that our estimates are based on the baseline risk distribution of Chilean patients and direct costs of the Chilean healthcare system, which may limit its application overseas. However, we argue that some relevant findings are valid across jurisdictions. For example, the independent relationship between risk, life expectancy and cost are both likely to hold beyond Chile. Although the higher risk is associated with lower life expectancy (negative relationship), both explain greater cost (positive relationship). Likewise, life expectancy and age are also significantly associated with cost, but in opposite direction. While the positive association between life expectancy and cost is expected, the negative association between age and cost seemed counterintuitive, since there was no clear reason to support the idea that between two patients whose time alive from diagnosis is the same, the younger is associated with fewer costs. To examine this finding, we explored the interaction between age and time alive. This analysis showed an estimate statistically significant (0.002; p-value < 0.0001) meaning that cost increases by 2% for every additional unit (year) of this interaction term. Thus, increases in age and time alive from diagnostic contribute to a higher cost.

Finally, we acknowledge that DM2 is a multifactorial disease, which interacts with many other conditions. Future research on modelling should link DM2 to other pre-diabetes disorders, such as insulin resistance or obesity, for a better characterization of the disease process. Furthermore, the precision of costs and outcomes estimates can also be improved by linking the diabetes model to specific models for each complication. Last, including other attributable complications, not considered in the UKPDS study, is also desirable for a more comprehensive representation. Although we could adapt an independent hypoglycaemia model to our DES model, other complications such as non-alcoholic fatty liver disease should be incorporated in future modelling exercises. In conclusion, DM2 is a highly prevalent condition that determines a significant expenditure on the health system and substantial health losses either in quantity or HRQoL. Although the control of cardiovascular risk factors and the metabolic control of the disease; both have an important impact on costs and outcomes, the main impact is achieved by postponing the age of onset of the disease.

## Supplementary Information

**Additional file 1:** Expected costs and outcomes of the outcome based subgroup analysis.

**Additional file 2:** Baseline characteristics of the microstimulated cohort and meta-analyzed utility values.

## Data Availability

All data generated or analyzed during this study are included in this published article [and its additional files.

## Abbreviations

BMI: Body Mass Index
CHS: Chilean Health Survey
CLP: Chilean pesos
DES: Discrete event simulation
DM2: Type 2 diabetes
DND: Diabetic nephropathy in dialysis
GLM: Generalized Linear Models
HbA1c: Haemoglobin A1c
HDL: High-density-lipoprotein cholesterol
HRQoL: Health-related quality of life
IHD: Ischemic Heart Disease
LDL: Low-density-lipoprotein cholesterol
MI: Myocardial infarction
QALY: Quality-adjusted life-years
RMST: Restricted mean survival time
SBP: Systolic Blood Pressure
UKPDS: United Kingdom Prospective Diabetes Study
