# Supplementary material for: Health and economic effects on patients with type 2 diabetes mellitus in the long run: predictions for the Chilean population
Source: Diabetol Metab Syndr. 2022 Oct 26;14:155. doi: 10.1186/s13098-022-00928-4 (PMC9598003; doi:10.1186/s13098-022-00928-4)
Supplement: Supplementary file 2 — Additional file 2: Baseline characteristics of the microstimulated cohort and meta-analyzed utility values. [file 13098_2022_928_MOESM2_ESM.docx]

**Additional file 2**

**Additional file 2: Table S1: Baseline characteristics of the microsimulated cohort**

|  | **Mean (SD)**  **Simulated cohort** | **Mean (SD)**  **Original Survey** |
| --- | --- | --- |
| Men (%) | 41.32 | 41.98 |
| Women (%) | 58.68 | 59.02 |
| Age (years) | 58.00 (11.55) | 57.59 (11.79) |
| BMI (Kg/m^2^) | 31.77 (5.38) | 31.96 (5.51) |
| Heart Rate (bpm) | 71.91 (9.39) | 71.87 (9.34) |
| HbA1_c_ (%) | 8.44 (2.12) | 8.38 (2.10) |
| LDL (mmol/L) | 2.92 (0.88) | 2.96 (0.87 |
| HDL (mmol/L) | 1.10 (0.29) | 1.11 (0.29) |
| BP (mmHg) | 138.2 (23.14) | 139.39 (23.61) |
| Current Smoker (%) | 30.90 | 29.50 |

Abbreviation: BMI, Body Mass Index; HbA1_c_, Glycated Hemoglobin A1_c_; LDL, Low-Density Lipoprotein; HDL, High-Density Lipoprotein; BP, Systolic Blood Pressure.

**Additional file 2: Table S2: Utility values**

**Random effect meta-analysis**

| Parameter | Point estimate | Sources |
| --- | --- | --- |
| Utility of Diabetes | 0.82 (0.004) | [1,2] |
| Disutility of Ischemic Heart Disease | 0.046 (0.026) | [3,4] |
| Disutility of Myocardial Infarction | 0.012 (0.007) | [3,5] |
| Disutility of Congestive Heart Failure | 0.053 (0.034) | [4,5] |
| Disutility of Stroke | 0.090 (0.069) | [3–6] |
| Disutility of Blindness | 0.030 (0.015) | [3,4] |
| Disutility of Hypoglycemia | 0.021 (0.001) | [7,8] |
| Disutility of Amputation | 0.122 (0.069) | [9] |
| Disutility of Second Amputation | 0.257 (0.105) | [5] |
| Disutility of Diabetic Nephropathy in Dialysis | 0.004 (0.016) | [5] |

**References**

1. Hunger M, Thorand B, Schunk M, Döring A, Menn P, Peters A, et al. Multimorbidity and health-related quality of life in the older population: Results from the German KORA-Age study. Health Qual Life Outcomes [Internet]. 2011;9(1):53. Available from: http://www.hqlo.com/content/9/1/53

2. Burström K, Johannesson M, Diderichsen F. Health-related quality of life by disease and socio-economic group in the general population in Sweden. Health Policy (New York). 2001;55(1):51–69.

3. Clarke P, Gray A, Holman R. Estimating Utility Values for Health States of Type 2 Diabetic Patients Using the EQ-5D (UKPDS 62). Med Decis Mak. 2002;22(4):340–9.

4. Lee WJ, Song KH, Noh JH, Choi YJ, Jo MW. Health-related quality of life using the EuroQol 5D questionnaire in Korean patients with type 2 diabetes. J Korean Med Sci. 2012;27(3):255–60.

5. Nauck MA, Buse JB, Mann JFE, Pocock S, Bosch-Traberg H, Frimer-Larsen H, et al. Health-related quality of life in people with type 2 diabetes participating in the LEADER trial. Diabetes, Obes Metab. 2019;21(3):525–32.

6. Solli O, Stavem K, Kristiansen IS. Health-related quality of life in diabetes: The associations of complications with EQ-5D scores. Health Qual Life Outcomes. 2010;8:1–8.

7. Shi L, Shao H, Zhao Y, Thomas NA. Is hypoglycemia fear independently associated with health-related quality of life? Health Qual Life Outcomes. 2014;12(1):1–9.

8. Polonsky WH, Thompson S, Wei W, Riddle MC, Chaudhari S, Jackson J, et al. Greater fear of hypoglycaemia with premixed insulin thanwith basal-bolus insulin glargine and glulisine: Patient-reported outcomes from a 60-week randomisedstudy. Diabetes, Obes Metab. 2014;16(11):1121–7.

9. Hayes A, Arima H, Woodward M, Chalmers J, Poulter N, Hamet P, et al. Changes in quality of life associated with complications of diabetes: Results from the ADVANCE study. Value Heal. 2016;19(1):36–41.
